# ACTH-independent Cushing’s syndrome due to ectopic endocrinologically functional adrenal tissue caused by a *GNAS* heterozygous mutation: a rare case of McCune–Albright syndrome accompanied by central amenorrhea and hypothyroidism: a case report and literature review

**DOI:** 10.3389/fendo.2022.934748

**Published:** 2022-07-25

**Authors:** Kai Takedani, Masahiro Yamamoto, Sayuri Tanaka, Shinichiro Ishihara, Takeshi Taketani, Keizo Kanasaki

**Affiliations:** ^1^ Internal Medicine 1, Shimane University Faculty of Medicine, Izumo, Japan; ^2^ Department of Pediatrics, Shimane University Faculty of Medicine, Izumo, Japan

**Keywords:** *GNAS* mutation, Cushing’s syndrome, McCune–Albright syndrome, ectopic adrenal tumor, fibrous dysplasia

## Abstract

In a small number of cases, the development of ectopic residual adrenal lesions during embryogenesis causing Cushing’s syndrome due to the production of excess cortisol has been reported. A 29-year-old woman was admitted to our hospital for fatigue and recent amenorrhea. Her plasma ACTH was <1.5 pg/mL, and her serum cortisol was 21.4 pg/mL after the 8 mg dexamethasone suppression test, revealing the presence of ACTH-independent Cushing’s syndrome; however, her bilateral adrenal glands were atrophied. Abdominal CT revealed a 40-mm round tumor on the right renal hilum and remarkably accumulated ^131^I-labelled adosterol. CT and bone scintigraphy showed that ^99m^Tc-methylene diphosphonate had accumulated in her dissymmetric skull at the right-frontoparietal region. The tumor on the right renal hilum was laparoscopically removed. Her cortisol levels rapidly decreased to below the normal range, and glucocorticoids were administered to rescue adrenal insufficiency. The resected tumor was yellowish in appearance and 4.5×3.0×2.8 cm in size. Immunohistochemical staining for SF-1, P450scc, CYP17A, CYP21A, and CYP11B1 indicated that this tumor produced cortisol. Exome sequencing analysis revealed that the *GNAS* heterozygous mutation (c.601C>T, p. Arg201Cys; accession number, NM_000516.5) was found in approximately 20% of the adrenal tumor sample. A mutation of *GNAS*, encoding the Gsα subunit that mediates GPCR signaling, causes the constitutive activation of adenylyl cyclase, resulting in hypersecretion of hormones regulated by the GPCR. *GNAS* mutation is one of the major genetic causes of cortisol-producing adrenal tumors independent of ACTH secretion. Considering the combination of *GNAS* mutation with one of the typical clinical triad characteristics, fibrous dysplasia of bone, we diagnosed this patient with McCune–Albright syndrome accompanied by ACTH-independent Cushing’s syndrome caused by an ectopic residual adrenal tumor due to *GNAS* mutation. This case highlights that *GNAS* involves a previously unknown pathological mechanism in which inhibition of the natural elimination of remnant tissue leads to ectopic endocrine hypersecretion.

## Introduction

Cushing’s syndrome, characterized by excess endogenous glucocorticoids, is a rare disease, with a prevalence of approximately 0.7-2.4 cases per million people per year. A total of 15%–20% of cases are adrenocorticotropic hormone (ACTH)-independent (1). A unilateral cortisol-producing tumor due to either adenoma or adrenocortical carcinoma is the primary cause (1); an ectopic cortisol-producing tumor in extra-adrenal tissue is an exceedingly uncommon aetiology for hypercortisolism.

McCune–Albright syndrome (MAS), a disease caused by a mutation of *GNAS*, predisposes individuals to hormonal hypersecretion from endocrine organs with G-protein-coupled receptors (GPCRs). However, there are only a few cases of MAS accompanied by ACTH-independent Cushing’s syndrome, which was limited in infancy, suggesting that ACTH-independent Cushing’s syndrome, especially in adults, is one of the rarest endocrine disorders in MAS (2). Here, we present the first case of ACTH-independent Cushing’s syndrome caused by an ectopic extra-adrenal cortisol-producing tumor at the renal hilum concomitant with *GNAS* mutation, which is crucial to the pathogenesis of MAS.

## Case description

A 29-year-old woman presenting with a six-month history of amenorrhea and general fatigue concomitant with lid oedema was admitted to our hospital. Spontaneous menstruation had started at the age of 13 years, and she had maintained a regular menstrual cycle since the age of 28. Her breasts, pubic hair, and axillary hair started to grow when she was approximately 13 years old. An annual medical check-up revealed hypertension four years previously, and she was treated with 40 mg amlodipine, 20 mg azilsartan, and 30 mg azosemide. Her legs and eyelids were oedematous approximately nine months before admission, and purpura emerged on the distal extremities. She had no significant medical history, including endocrine dysfunction, bone disease, or intellectual disability.

Her blood pressure was 142/104 mmHg. Her weight was 51.7 kg, her height was 154 cm (body mass index of 21.8 kg/m^2^), and her right and left grip strength was 16.8 and 13.3 kg, respectively. She had a typical moon-face, central obesity with petechiae, and thin limbs; however, she had no other cushingoid features, such as red abdominal striae or a buffalo hump. Oedema was observed in the bilateral eyelids and lower extremities; the latter was accompanied by subcutaneous bleeding. Physical examination revealed that the right-frontoparietal region of her cranium was asymmetrically deformed, exhibiting bulging; however, cafe-au-lait spots were not observed.

**Figure 1:**
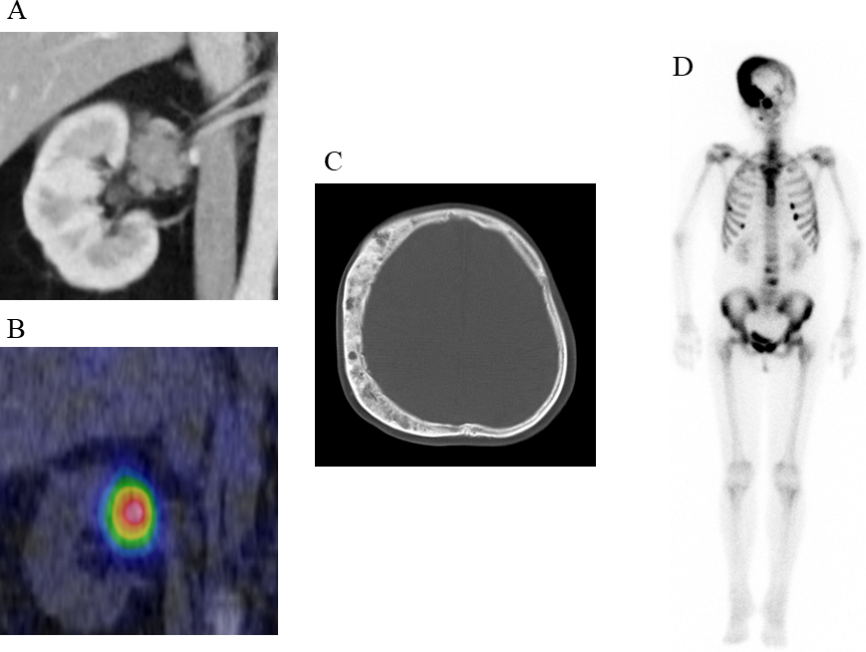
Clinical images of the patient **(A)** Enhanced CT scan showing a 40-mm round tumor with a relatively reduced contrast effect in the right renal hilum. **(B)** Remarkable ^131^I-labelled adosterol accumulation in the tumor but not in the adrenal glands. **(C)** CT scan showing dissymmetric bone thickening with a “ground-glass” appearance, which consisted of cystic or solid mass at right-frontoparietal region of the temporal bone. **(D)** ^99m^Tc-methylene diphosphonate (MDP) accumulation in the skull lesion.

A laboratory examination showed that most of the pituitary hormones secreted from the anterior lobe were at low levels, and corresponding target hormones, except for cortisol, were decreased: ACTH 3.4 pg/mL, cortisol 26.8 μg/dL; thyroid-stimulating hormone (TSH) 0.09 µIU/mL, free thyroxine (FT_4_) 0.65 ng/dL, and free triiodothyronine (FT_3_) 1.25 pg/mL; luteinizing hormone (LH) 4.6 mIU/mL, follicle-stimulating hormone (FSH) 6.1 mIU/mL, and E_2_ 21 pg/mL; growth hormone (GH) 0.1 ng/mL, and insulin-like growth factor 1 (IGF-1) 81 ng/mL; and prolactin (PRL) 16.5 ng/dL. Thyrotropin-releasing hormone (TRH), growth hormone releasing peptide-2 (GHRP-2), and gonadotropin-releasing hormone (GnRH) provocation tests revealed poor responses of TSH and GH and low elevations of PRL, LH, and FSH, indicating that she suffered from central hypopituitarism. Notably, the urinary cortisol level was higher than the normal range (716 μg/day), which was accompanied by hypercortisolaemia with loss of circadian rhythm. The serum cortisol level after the administration of 8 mg dexamethasone was not suppressed (21.4 μg/dL), indicating that hypercortisolaemia was caused by ACTH-independent Cushing’s syndrome. However, abdominal computed tomography (CT) showed that both her adrenal glands were atrophied. No apparent hormonal dysfunction of the other adrenal gland was observed (plasma aldosterone 93 pg/mL, plasma renin activity 1.0 ng/mL/hr, serum dehydroepiandrosterone sulphate (DHEA-S) 180 ng/mL, and urinary concentrations of the catecholamine metabolites metanephrine and normetanephrine 0.05 and 0.14 mg/day, respectively). Coincidentally, an enhanced CT scan showed a 40-mm round tumor on the right renal hilum (**Figure 1A**); the tumor remarkably accumulated ^131^I-labelled adosterol but not in the adrenal glands (**Figure 1B**). According to these findings, we considered that this extra-adrenal tumor ectopically produced cortisol independent of ACTH secretion. In addition, a head CT scan showed dissymmetric bone thickening with a “ground-glass” appearance, which consisted of a cystic or solid mass at right-frontoparietal region of the temporal bone (**Figure 1C**). Bone scintigraphy showed that ^99m^Tc-methylene diphosphonate (MDP) accumulated in multiple bones, including skull lesions (**Figure 1D**), suggesting that these bone features were caused by fibrous dysplasia.

**Figure 2:**
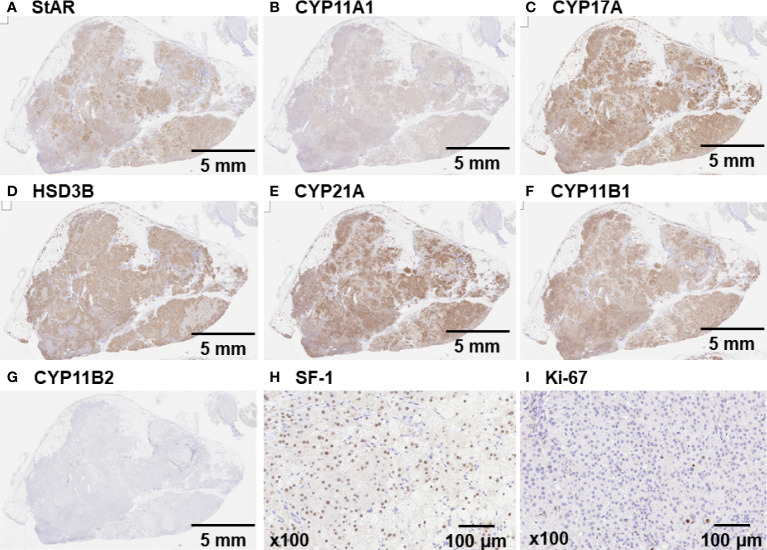
Immunohistochemical staining of the tumor on the right renal hilum Immunohistochemical staining confirmed a pituitary adenoma that was markedly positive for **(A)** StAR, **(B)** CYP11A1, **(C)** CYP17A, **(D)** HSD3B, **(E)** CYP21A, **(F)** CYP11B1, and **(H)** SF-1. **(G and I)** Staining for CYP11B2 and Ki-67 was negative.

The tumor on the right renal hilum was laparoscopically removed. The resected tumor was yellowish in appearance, 16 g in weight, and 4.5×3.0×2.8 cm in size. Positive immunohistochemical staining for StAR, CYP11A1, CYP17A, HSD3B, CYP21A, CYP11B1, and SF-1 (**Figures 2A**–**F**, **H**) indicated that the tumor was capable of producing cortisol from cholesterol, a source reagent, *via* an authentic enzymatic reaction. In contrast, staining for CYP11B2 and Ki-67 was negative (**Figures 2G**, **I**). An exome sequencing analysis using the Illumina platform revealed that a *GNAS* heterozygous mutation (c.601C>T, p. Arg201Cys; accession number, NM_000516.5) was found in approximately 20% of the adrenal tumor sample.

After resection, the patient’s cortisol levels rapidly decreased to below the normal range, and glucocorticoids were administered to rescue adrenal insufficiency. Antihypertensive medication was not required to control her blood pressure. Three months after surgery, the Cushingoid features disappeared, she spontaneously resumed menstruation, and levothyroxine was discontinued. A year later, her cortisol levels remained within the normal range without replacement therapy. Finally, we diagnosed her with MAS because the coexisting findings of fibrous dysplasia and ACTH-independent hypercortisolaemia caused by the ectopic tumor on the right renal hilum could be explained by only one cause, *GNAS* mutation.

## Discussion

The patient underwent the surgical removal of the extra-adrenal tumor on the right renal hilum, which possessed a GNAS heterozygous mutation. Tumor resection induced the normalization of ACTH-independent hypercortisolaemia and the resolution of hypopituitarism, including hypogonadotropic amenorrhea and central hypothyroidism.

One of the unique clinical features in this case was that the tumor on the right renal hilum produced cortisol. Adrenal foetal zone tissue that detaches in the neonatal period and migrates through the urogenital tract can result in residual tissue that forms tumors on the retroperitoneum, testis, broad ligament, ovaries, and inguinal region, including the kidneys, and this residual tissue is considered ectopic adrenal tissue (3, 4). Only 1% of these lesions are observed in adults due to atrophic changes during tissue development, although they are observed in 50% of newborn infants (3), and most cases are asymptomatic (5). However, in our case, the tumor on the right renal hilum possessed all of the adrenal-specific biosynthetic enzymes at each step in the conversion of cholesterol to cortisol, and the gene expression of the tumor is known to be regulated by steroidogenic factor-1:SF-1 (6). In addition, ^131^I-labelled adosterol accumulated in this tumor. These observations were sufficient to consider that this tumor was an ectopic adrenal gland. Moreover, this ectopic lesion was an endocrinologically functional adrenal gland that was responsible for ACTH-independent Cushing’s syndrome because tumor resection induced the resolution of hypercortisolaemia and led to adrenal failure. Previous reports describing ectopic adrenal tumors that secrete adrenocortical hormones are summarized in **Table 1** (3, 5, 7–19). Among the fifteen previous cases, these tumors were most commonly found in those aged less than 40 years old (11 cases), and the majority produced cortisol (14 cases) and were classified as adenoma or hyperplasia (10 cases). The most common tumor site was around a kidney, such as the renal hilum (6 cases), and all these tumors were benign. The histopathological characteristics and steroidogenic enzyme profile of ectopic cortisol-producing adrenocortical adenomas are similar to those of monotopic adrenocortical adenomas and those in our case (16). A review of the literature supported our diagnosis of a functional ectopic adrenal tumor at the renal hilum that caused ACTH-independent Cushing’s syndrome.

**Table 1:** Reported cases of Cushing’s syndrome due to ectopic adrenal tissue.

No.	Publicationyear	Age/Sex	Size of tumor	Location of tumor	Hormonal levels(before treatment)	Histology	Immuno-stainingfindings	mRNA expression	Genetic mutation	Remarks
1	1963 (7)	40/M	7 cm	inferior pole of the left kidney	plasma 17-OHCS 10.1 μg/dL	hyperplasia	NA	NA	NA	2 years after bilateral adrenalectomy No recurrence (but died of another disease 17 months later)
2	1966 (8)	59/M	7.5 cm	above the upper pole of the right kidney	urinary 17-OHCS 29 mg/day urinary 17-KS 5 mg/day	carcinoma	NA	NA	NA	4 months after bilateral adrenalectomy
3	1969 (9)	26/F	15 cm	upper pole of the left kidney	17-KS 159 mg/day	carcinoma	NA	NA	NA	No recurrence for 15 months
4	1972 (10)	34/F	1.5 cm and 1.2 cm	each para- ovarian area	Cor 15 μg/dL	adrenocortical tissue	NA	NA	NA	After bilateral adrenalectomy and external irradiation of the pituitary gland
5	1981 (11)	23/F	18 cm	liver	Cor 26 μg/dL	(probably) malignant	Cor (+) testosterone (+)	NA	NA	Died of a pulmonary embolism before treatment
6	1985 (12)	21/F	12 cm	liver	Cor 41 μg/dL UFC 1627 μg/day	carcinoma with low malignancy potential (difficult to determine whether this tumor was benign or malignant)	NA	NA	NA	No recurrence for 9 months Resection after ketoconazole therapy
7	1998 (5)	33/F	3.0 cm	adjacent to the left adrenal gland	Cor 14 μg/dL ACTH 6.1 pg/mL UFC 78 μg/day	adenoma	NA	NA	NA	14 years after left adrenalectomy
8	2000 (13)	63/F	3.5 cm	left renal hilum	Cor 25 μg/dL (after 1 mg Dex) ACTH < 1.0 pg/mL UFC 130 μg/day (after 2 mg Dex)	adenoma	NA	NA	NA	No recurrence for 9 months
9	2010 (14)	35/F	3.8 cm	left renal hilum	Cor 25 μg/dL ACTH < 5.0 pg/mL UFC 645 μg/day	adenoma	CYP17A1 (+) CYP21 (+)	CYB11B1 (+) (higher than normal adrenal level)	*PRKAR1A* (-) *PDE8B* (-) *PDE11A* (-)	3 months after bilateral adrenalectomy
10	2012 (15)	38/M	4.0 cm	left renal hilum	Cor 27 μg/dL ACTH < 1.0 pg/mL UFC 1927 μg/day	adenoma	NA	NA	NA	No recurrence
11	2014 (16)	53/F	1) 3.5 cm 2) 2.7 cm	1, 2) left renal hilum	1) NA 2) Cor 18 μg/dL ACTH < 5.0 pg/mL UFC 159 μg/day	1, 2) adenoma	1) NA 2) Melan-A (+) HSD3B2 (+) CYP17A1 (+)	1) NA 2) CYP11B1 (+) CYP11B2 (+) CYP17A1 (+) HSD3B2 (+) (similar to adrenal CPA)	1, 2) NA	1) First diagnosis 2) 2 years after first surgery, recurrence (+)
12	2016 (17)	37/F	3.4 cm	right renal sinus	NA (Cor was higher than normal)	adenoma	synaptophysin (+) CD56 (+), vimentin (+) Ki-67 (2%) calretinin (+) inhibin-α (+) chromogranin A (-) CD117 (-), CD10 (-) CK7 (-), EMA (-) CK-pan (-), melan-A (-) pax-8(±)	NA	NA	No recurrence for a month
13	2018 (3)	18/F	3.0 cm	left renal hilum	Cor 21 μg/dL ACTH 1.3 pg/mL UFC 1824 μg/day	adenoma	inhibition (+) melan-A (+) synaptophysin (+) vimentin (+) AE1/AE3 (+) HMB45 (±) CD34 (+, angiographic) NSE (-), CgA (-)	NA	NA	No recurrence for 12 months
14	2018 (18)	46/M	3.6 cm	right renal hilum	Cor 37 μg/dL ACTH < 1.0 pg/mL UFC 159 μg/day	adenoma and myelolipoma metaplasia	KI-67 (3%)	NA	NA	No recurrence for 6 months
15	2018 (19)	21/F	16 cm (CT scan)	liver	Cor 27 μg/dL	carcinoma	CD 56 (+), HEP 1 (+) NSE (+) (liver biopsy)	NA	NA	Referred to another hospital for surgical therapy,
16	our case	29/F	4.0 cm	right renal hilum	Cor 26.8 μg/dL, ACTH 3.4 pg/mL, UFC 716 μg/day	cor-producing lesion (benign)	StAR (+) CYP11A1 (+) CYP17A (+) HSD3B (+) CYP21A (+) CYP11B1 (+) SF-1 (+) CYP11B2 (-)	NA	*GNAS* (+) c.601C>T, p.Arg201Cys	presence of fibrous dysplasia

NA, not available.

17-OHCS, 17-hydroxycorticosteroid; 17-KS, 17-ketosteroid; Cor, cortisol; UFC, urinary free cortisol; ACTH, adrenocorticotropic hormone; Dex, dexamethasone; CPA, cortisol-producing adrenocortical adenoma.

The most crucial and novel finding is that we detected for the first time the *GNAS* mutation, encoding the Gsα subunit that mediates GPCR signalling, from this endocrinologically functional ectopic tumor. *GNAS* mutation causes the constitutive activation of adenylyl cyclase, which activates cAMP-dependent protein kinase A (PKA), leading to the hyperproduction of cortisol through the acceleration of the cAMP response element-binding protein CREB (20). Indeed, the *GNAS* mutation was detected in 16.9% out of 65 cases of ACTH-independent Cushing’s syndrome (21), suggesting that this mutation is one of the causal molecular pathogeneses for cortisol-producing adrenocortical adenoma. We considered that residual adrenal tissue, which possessed the *GNAS* mutation occurring in early development, autonomously secreted excessive cortisol through an increase in cAMP due to the constitutive activation of Gsα, resulting in ACTH-independent Cushing’s syndrome in this patient.

One of the more recognized diseases associated with *GNAS* mutation is MAS. MAS is classically defined as a clinical triad involving fibrous dysplasia of bone, café-au-lait spots, and precocious puberty (2). MAS is often diagnosed according to the presence of two or more of these typical features. However, it is now recognized that those phenotypes are more complex. The prevalence of major findings, fibrous dysplasia of bone, café-au-lait spots, and precocious puberty (female) were 98%, 66%, and 50%, respectively (22), suggesting that patients with MAS who have typical triad are not frequent. If patients have only fibrous dysplasia, which is the most common feature in MAS (23), identification of *GNAS* mutation by genetic testing is needed to establish the diagnosis (24). This suggests that the detection of this underlying genetic pathogenesis along with MAS-related symptoms is essential in establishing a definitive diagnosis of MAS. The Gsα protein, which is encoded by the *GNAS* gene, is a ubiquitous cellular component. If Gsα mutation occurs in the GPCR signalling pathway of the endocrine system, such as in LH, TSH, GnRH, ACTH, or bone tissue, it causes constant activation of intracellular signal transduction and results in precocious puberty, thyrotoxicosis, ACTH-independent Cushing’s syndrome, acromegaly, or FGF23-related hypophosphatemic rickets/osteomalacia (2). Such hypersecretion from endocrine organs with GPCRs is known to be associated with MAS (22). In this case, our patient did not have a café-au-lait spot and did not suffer from precocious puberty. However, we detected ACTH-independent hypercortisolaemia accompanied by *GNAS* gene mutation in the extra-adrenal tumor as well as fibrous dysplasia with typical findings on CT and bone scintigraphy. MAS is the most probable single aetiology that accounts for the observed findings in both the bone and ectopic adrenal glands.

Hypopituitarism, which was observed in this patient, causing conditions such as amenorrhea and hypothyroidism is not a typical clinical feature of MAS. Elevated cortisol is known to act on the hypothalamus and reduce the basal gonadotropins level, resulting in hypogonadotropic hypogonadism. Hypercortisolism suppresses TRH and TSH release, leading to central hypothyroidism. Hypopituitarism in the patient spontaneously recovered after the removal of the renal hilum tumor, indicating that hyposecretion of anterior pituitary hormones, except for ACTH, was caused by secondary hypopituitarism, not primary hypopituitarism.

This is the first report of an ectopic endocrinologically functional adrenal tumor due to a *GNAS* heterozygous mutation causing ACTH-independent Cushing’s syndrome. The patient in this case had concomitant typical fibrous dysplasia; thus, we supposed that the clinical symptoms were caused by MAS. *GNAS*-related GPCRs are widely distributed in the body; thus, the clinical symptoms of MAS caused by the somatic mosaic phenotype vary widely. This case highlights that *GNAS* is associated with a previously unknown pathological mechanism in which inhibition of the natural elimination of remnant tissue leads to ectopic endocrine hypersecretion.

## Data availability statement

The original contributions presented in the study are included in the article/supplementary material. Further inquiries can be directed to the corresponding author.

## Ethics statement

Written informed consent was obtained from the individual(s) for the publication of any potentially identifiable images or data included in this article.

## Author contributions

KT wrote the first draft of the manuscript. MY contributed to the writing of the manuscript. KT, ST, SI, and TT made contributions to the acquisition of the clinical data. MY and KK made critical revisions. All authors contributed to the article and approved the submitted version.

## Conflict of interest

The authors declare that the research was conducted in the absence of any commercial or financial relationships that could be construed as a potential conflict of interest.

## Publisher’s note

All claims expressed in this article are solely those of the authors and do not necessarily represent those of their affiliated organizations, or those of the publisher, the editors and the reviewers. Any product that may be evaluated in this article, or claim that may be made by its manufacturer, is not guaranteed or endorsed by the publisher.
